# Calibration-Free 3D Indoor Positioning Algorithms Based on DNN and DIFF

**DOI:** 10.3390/s22155891

**Published:** 2022-08-07

**Authors:** Jingmin Yang, Shanghui Deng, Li Xu, Wenjie Zhang

**Affiliations:** 1 School of Computer Science, Minnan Normal University, Zhangzhou 363000, China; 2 Key Laboratory of Data Science and Intelligence Application, Minnan Normal University, Zhangzhou 363000, China; 3 Fujian Provincial Key Laboratory of Network Security and Cryptology, Fujian Normal University, Fuzhou 350007, China

**Keywords:** calibration-free, deep denoising autoencoder (DDAE), fingerprint database, signal strength difference (DIFF), 3D indoor positioning

## Abstract

The heterogeneity of wireless receiving devices, co-channel interference, and multi-path effect make the received signal strength indication (RSSI) of Wi-Fi fluctuate greatly, which seriously degrades the RSSI-based positioning accuracy. Signal strength difference (DIFF), a calibration-free solution for handling the received signal strength variance between diverse devices, can effectively reduce the negative impact of signal fluctuation. However, DIFF also leads to the explosion of the RSSI data dimension, expanding the number of dimensions from *m* to $C_{m}^{2}$, which reduces the positioning efficiency. To this end, we design a data hierarchical processing strategy based on a building-floor-specific location, which effectively improves the efficiency of high-dimensional data processing. Moreover, based on a deep neural network (DNN), we design three different positioning algorithms for multi-building, multi-floor, and specific-location respectively, extending the indoor positioning from the single plane to three dimensions. Specifically, in the stage of data preprocessing, we first create the original RSSI database. Next, we create the optimized RSSI database by identifying and deleting the unavailable data in the RSSI database. Finally, we perform DIFF processing on the optimized RSSI database to create the DIFF database. In the stage of positioning, firstly, we design an improved multi-building positioning algorithm based on a denoising autoencoder (DAE). Secondly, we design an enhanced DNN for multi-floor positioning. Finally, the newly deep denoising autoencoder (DDAE) used for specific location positioning is proposed. The experimental results show that the proposed algorithms have better positioning efficiency and accuracy compared with the traditional machine learning algorithms and the current advanced deep learning algorithms.

## 1. Introduction

With the widespread popularity of mobile terminal devices and the rapid development of Internet technologies, smart devices play an increasingly important role in the field of smart cities, providing people with various smart services, such as smart homes, smart healthcare, smart transportation systems, etc. [1,2,3]. With the wide application of GPS, GLONASS, BDS, GALILEO, and other global positioning systems, outdoor location-based services (LBS) play an important role in people’s daily life, such as emergency services, employee management, and vehicle tracking [4,5]. In recent years, with the rapid growth of mobile devices, indoor positioning has become more and more important, such as the indication of vacant parking spaces in indoor parking lots, the location monitoring of infants in hospitals, and the route guidance for customers in large shopping malls, etc. [6,7,8]. However, indoor positioning still faces the challenge of low positioning accuracy. Therefore, indoor positioning is still an issue worth studying.

The common technologies for indoor positioning include radio frequency identification device (RFID) [9,10], ZigBee [11,12], bluetooth [13], ultra-wideband (UWB) [14], Wi-Fi [15], etc. RFID technology has low power consumption and a wide range of applications, but it is prone to interference, resulting in low positioning accuracy. ZigBee is widely used in wireless sensor networks. But this technology is not available on commonly used devices, such as mobile phones. Therefore, additional equipment is required, which increases extra costs. Bluetooth has the advantages of high throughput, wide receiving range, and low energy consumption. But it is prone to noise, resulting in low positioning accuracy. UWB has high accuracy with strong anti-interference to multipath effects, but has a relatively short range and requires additional hardware support, resulting in high costs. A wireless local area network (WLAN) is developed based on the IEEE 802.11 series standards issued by the Institute of Electrical and Electronics Engineers (IEEE). It uses high-frequency wireless signals as the transmission medium, such as wireless electromagnetic waves in the 2.4 GHz or 5 GHz band. In 2018, a textbook titled “Wireless network technology: principle, experiment, and network design”, written by us, states that WLAN has the characteristics of flexible use, convenient expansion, effective costs, and simple installation [16]. Meanwhile, with the advancement of wireless communication technologies and the development of indoor communication requirements, WLAN is developing rapidly. On the other hand, compared with the aforementioned indoor positioning technologies, Wi-Fi-supported devices are diverse and inexpensive, and Wi-Fi signals are easy to be measured. These advantages make Wi-Fi networks widely used for indoor positioning [17].

The traditional indoor positioning methods such as angle of arrival (AoA), time of arrival (ToA), and time difference of arrival (TDoA) all require line-of-sight (LoS) measurements. The process of collecting fingerprint signals and correlating indoor locations without measuring LoS has become one of the prevalent research methods in the field of indoor positioning [18]. Fingerprint-based positioning methods are usually divided into two stages, the offline and the online [19]. In the offline phase, the RSSI of different reference locations is precisely measured, and a fingerprint database with RSSI and location mapping is created. In the online phase, specific coordinates are calculated by comparing the RSSI measured at the point to be located with the fingerprint database obtained in the offline phase.

In most cases, the RSSI-based Wi-Fi fingerprint positioning methods have high feasibility [20]. However, the current RSSI-based Wi-Fi fingerprint positioning methods still have the following issues that affect the positioning accuracy: (1) When the Wi-Fi signal is blocked by buildings, walls, and other objects during the propagation process, the multi-path effect is more likely to occur. (2) With the widespread popularity of mobile terminals, The heterogeneity of wireless devices leads to large fluctuations in the RSSI value of the same anchor point received at the same position. (3) At present, the commonly used Wi-Fi frequency band is 2.4 GHz. When multiple devices work at the same time, the co-channel interference increases the noise of the RSSI value received by the devices. We analyze these issues in detail in a study published in 2020 and note that these issues severely degrade RSSI-based positioning accuracy [21].

To address the above issues, we propose calibration-free indoor positioning algorithms based on DNN and DIFF. Compared with the existing Wi-Fi fingerprint database positioning methods, the main contributions of our work are summarized as follows.

(1) Aiming at the issue of positioning inefficiency caused by the data dimension explosion of DIFF, we design a data hierarchical processing strategy based on building-floor-specific location, which effectively improves the efficiency of high-dimensional data processing. Correspondingly, we create the original RSSI database, the optimized RSSI database, and the DIFF database. The optimized RSSI database is for multi-building positioning, and the DIFF database is for multi-floor and specific-location positioning. The update and processing of these three databases are performed in an asynchronous mode.

(2) The proposed DNN-based algorithms extend the indoor location from a single plane to three dimensions, namely, multi-building, multi-floor and specific locations. We design an improved DAE algorithm based on optimized RSSI for multi-building positioning, which improves the positioning accuracy up to 100%. On this basis, We design an enhanced DNN algorithm for multi-floor positioning. Finally, a newly designed specific-location positioning algorithm called DDAE is proposed. The DDAE consists of an optimized DAE and an enhanced DNN. The key idea is to utilize the DAE to remove redundant information and reduce the dimensions of input data DIFF while retaining the key features. Then, the dimensionality-reduced features are used as the input for the next part of the enhanced DNN. We evaluate our algorithms on the UJIIndoorLoc dataset compared with various baseline machine learning algorithms and deep learning algorithms. The results show that the proposed algorithms have better positioning efficiency, and the average positioning error at the specific position is also the smallest without extra calibration.

The rest of this paper is organized as follows. Section 2 reviews the related work of indoor positioning. Section 3 introduces the 3D indoor positioning algorithms. Section 4 presents the experimental preparation, including the RSSI-based data set and its optimization, the processing of DIFF, the establishment of the calibration-free DIFF database, and the evaluation indicators for algorithms. Section 5 studies the optimization of the algorithms, as well as evaluates the performance of the proposed algorithms. Finally, Section 6 summarizes the whole study work and points out the future research work.

## 2. Related Work

In general, according to whether the distance is measured, the indoor positioning methods can be divided into two types:ranging-based and non-ranging-based [22]. The ranging-based positioning methods have been widely used in outdoor environments, such as ToA, TDoA, AoA, etc. Meanwhile, RSSI-based fingerprint positioning is a commonly used indoor positioning method without ranging [23]. This method includes two stages: the offline fingerprint collection stage and the online fingerprint positioning stage. The purpose of the offline collection fingerprint stage is to build a database that matches specific location coordinates and RSSI fingerprint features. Specifically, at each collection point, by collecting the RSSI values of different access points (APs), the corresponding relationship between the RSSI features and the coordinates of the collection point can be established. In the online positioning phase, the RSSI of the to-be-located point is collected as the input data of the positioning algorithm, and the specific coordinates of the to-be-located point are calculated by the positioning algorithm.

In specific practice, the heterogeneity of wireless receiving devices, co-channel interference, and multi-path effect make the RSSI of Wi-Fi fluctuate greatly, which seriously degrades the RSSI-based positioning accuracy and robustness [21]. At present, there are two main methods to solve the problem of RSSI fingerprint fluctuation: calibration methods and calibration-free methods. The calibration methods establish the RSSI relationship model of the reference devices and the to-be-located mobile terminal at the same location in the offline stage. In the online phase, the RSSI relationship model is used to compensate for the RSSI measured of the to-be-located mobile terminal. The calibration methods have obtained many research results. The most significant advantage of calibration methods is that it is straightforward and effective. However, these methods also have some problems, such as higher delay in the calibration process [24]. The calibration-free methods optimize the original RSSI fingerprint to obtain a new fingerprint with better robustness. For example, the hyperbolic location fingerprinting (HLF) method proposed by Kjaergaard [25] uses the pairwise ratio of the collected RSSI as new fingerprints. The HLF achieves better localization accuracy but also increases the computational complexity of fingerprint comparison. In the reference [26], a calibration-free method of fingerprints called DIFF is proposed. The DIFF constructs a new fingerprint database by extracting the pairwise signal strength differences from the raw RSSI values. Through experimental comparison, the performance of the DIFF is better than that of the HLF.

A large number of RSSI-based machine learning methods have been proposed for indoor positioning, such as support vector machines (SVM) [27], k-nearest neighbors (KNN) [28], random forests (RF) [29], decision trees (DT) [30], and Gaussian Naïve-Bayes (GNB) [31]. In recent years, with the rapid development of deep learning, more and more deep learning technologies are applied to the field of indoor positioning. In 2017, Nowicki et al. designed a SAEDNN based on stacked autoencoder (SAE) and DNN for floor positioning [32]. In 2018, Jang et al. propose a CNN-based classifier for multi-building positioning [33]. These experiments are conducted on the UJIIndoor dataset [28]. In 2018, Kim et al. propose an improved model called SAEDNN, which reduces the number of nodes in the last layer [34]. In 2019, Song et al. propose an improved model called CNNLoc, which is proved to be superior to the above algorithms [35]. However, the above methods do not take into account the positioning of specific locations within a given floor, which is a more challenging issue. In 2021, Qin et al. propose a CDAE-CNN-based Wi-Fi fingerprint location algorithm called Ccpos, which uses the K-means algorithm to segment the dataset, and then uses the CDAE-CNN network for specific location prediction [36]. In 2022, Wang et al. designed a novel DNN-based indoor positioning framework called CHISEL, which combines a convolutional encoder and a CNN classifier [37]. Jaehoon Cha et al. propose a hierarchical auxiliary deep neural network called HADNN, which uses a continuous feedforward network to identify buildings and estimate the floor coordinates [38].

The above research work has achieved amazing findings, but the influence of RSSI fluctuation is not considered separately. In other words, the above work unconsciously deals with this problem by using the technologies of deep learning. Inspired by the research work of the above-mentioned outstanding scholars, we combine the DIFF and deep learning to design new algorithms that extend indoor localization from plane positioning to 3D positioning. In the following sections, the key idea and specific design of these algorithms are discussed in detail.

## 3. Methodology

This section first introduces the overall structural design of the positioning system and then presents the theoretical basis and the design of the multi-building positioning model, multi-floor positioning model, and specific-location positioning model in detail.

### 3.1. Positioning System Architecture

**Figure 1:**
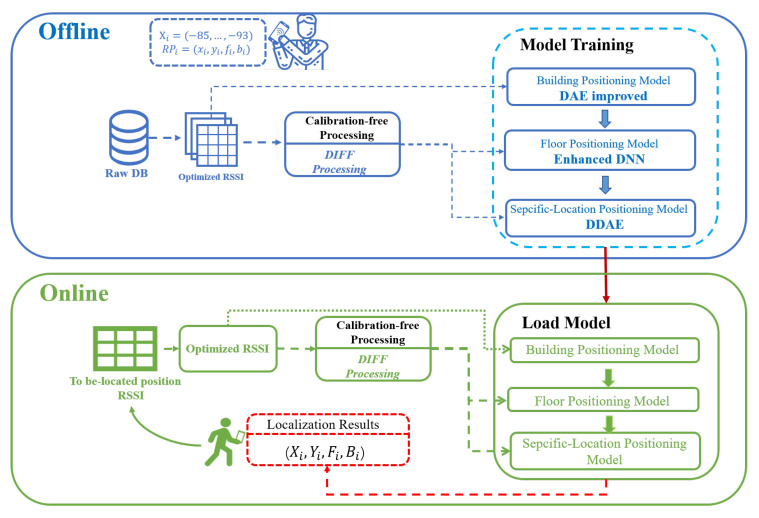
System Architecture.

The overall positioning system architecture proposed in this paper is shown in Figure 1.

The indoor positioning process is mainly composed of the offline training phase and the online positioning phase. In the offline training phase, firstly, we collect RSSI and build the original RSSI fingerprint database. Secondly, we identify and delete the unavailable data in the RSSI database, and then create the calibration-free DIFF fingerprint database based on the optimized RSSI database. The update of the RSSI database, the optimization of RSSI data, and the update of the DIFF database adopt asynchronous operation mode and have no impact on each other. Finally, we use the optimized RSSI database to train the multi-building positioning model, and the DIFF database to train the multi-floor positioning model and specific-location positioning model, respectively. In the online positioning stage, the RSSI fingerprint measured at the to-be-located position is optimized first, and then converted into the calibration-free DIFF. Then, through the trained positioning algorithms, the 3D coordinate values of the building-floor-specific location are calculated, respectively, and returned to the requester.

### 3.2. Multi-Building Positioning Model

Autoencoder (AE) is a kind of unsupervised learning artificial neural network (ANN). Its main function is to perform representation learning on the input information, remove redundant information, and retain the key features. AE is widely used in dimensionality reduction and anomaly detection [39]. AE consists of the encoder and the decoder. Given high-dimensional input space *X*, the low-dimensional feature space *F* is obtained through the encoder *f*, and then the high-dimensional space $\hat{X}$ is restored through the decoder g. The AE solves the mapping *f* and *g* by minimizing the reconstruction error of the input feature *X* and the restored feature $\hat{X}$, that is,

(1) $$\begin{array}{cc} & f:X\to F \end{array}$$

(2) $$\begin{array}{cc} & g:F\to \hat{X} \end{array}$$

(3) $$\begin{array}{cc} & f,g=arg\underset{f,g}{min}||X-{g\left[f\left(X\right)\right]||}^{2} \end{array}$$

**Figure 2:**
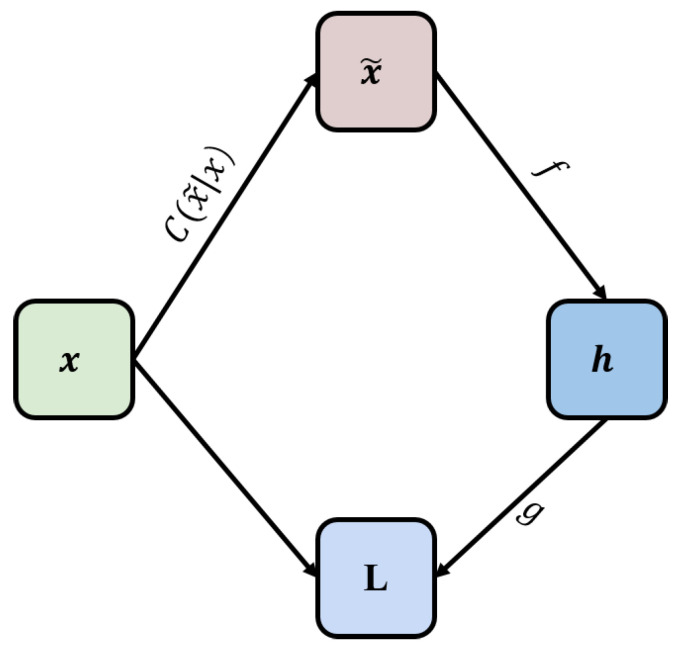
DAE Network.

Affected by problems such as model complexity, the training set data volume, and data noise, the initial model obtained through AE often has the risk of over-fitting. The DAE solves this problem effectively by reconstructing the input data with noise [40]. The DAE model is shown in Figure 2.

Figure 2 introduces a noise process $C(\tilde{x}|x)$. $C(\tilde{x}|x)$ is a conditional probability, which represents the probability of generating noise samples $\tilde{x}$ given the data sample *x*. DAE is trained to reconstruct the original sample x from the noise-added $\tilde{x}$ by minimizing the loss function $L=-logp_{decoder}\left(x|h=f\left(\tilde{x}\right)\right)$, where $\tilde{x}$ is the sample obtained from the initial sample *x* after the noise processing $C(\tilde{x}|x)$. Usually, the distribution function $p_{decoder}$ is the distribution of the DAE neural network parameters. DAE learns the reconstruction distribution $p_{decoder}$ from the training data pair $\left(x,\tilde{x}\right)$ according to the following process:

1. Randomly take a training sample *x* from the training set.
2. Randomly take a noise sample $\tilde{x}$ from the noise process $C(\tilde{x}|x)$.
3. Estimate the reconstruction distribution $p_{decoder}\left(x|\tilde{x}\right)=p_{decoder}\left(x|h\right)$ of the ADE by using the data pair $\left(x,\tilde{x}\right)$ as training samples.

In general, we can use the gradient descent method to minimize the approximate solution of the negative log-likelihood function $-logp_{decoder}\left(x|h\right)$. Therefore, DAE can be expressed as a random gradient descent function under the following expectations, where ${\hat{p}}_{data}$ is the distribution of the training data:

(4) $$\begin{array}{c} -E_{x∼{\hat{p}}_{data}\left(x\right)}E_{\left(\tilde{x}∼C\right)}\left(\tilde{x}|x\right)logp_{decoder}\left(x|h=f\left(\tilde{x}\right)\right) \end{array}$$

**Figure 3:**
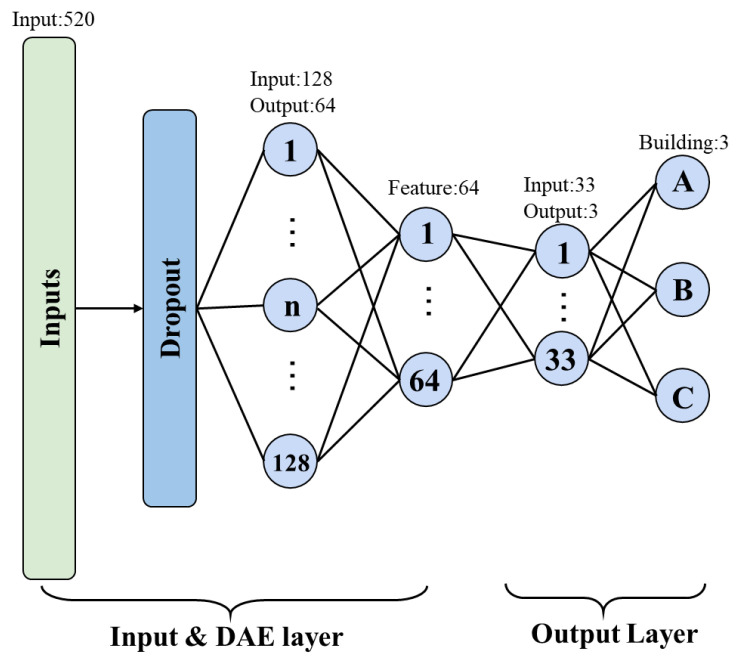
Multi-building positioning Model.

When adopting the hierarchical positioning strategy, the classification accuracy of multi-building is the first key issue. Because the wrong classification of the building makes subsequent multi-floor localization and specific location localization meaningless. After analysis, we find that the distance between buildings is relatively long, and the difference in Wi-Fi signals in each building is obvious. Therefore, we use the optimized RSSI as the input value of the model and design an improved DAE to Increase the anti-noise ability of the model. After the training of DAE, the denoised features are connected to a fully connected hidden layer, and finally the buildings are classified by the softmax layer. The processing process is shown in Figure 3.

### 3.3. Multi-Floor Positioning Model

**Figure 4:**
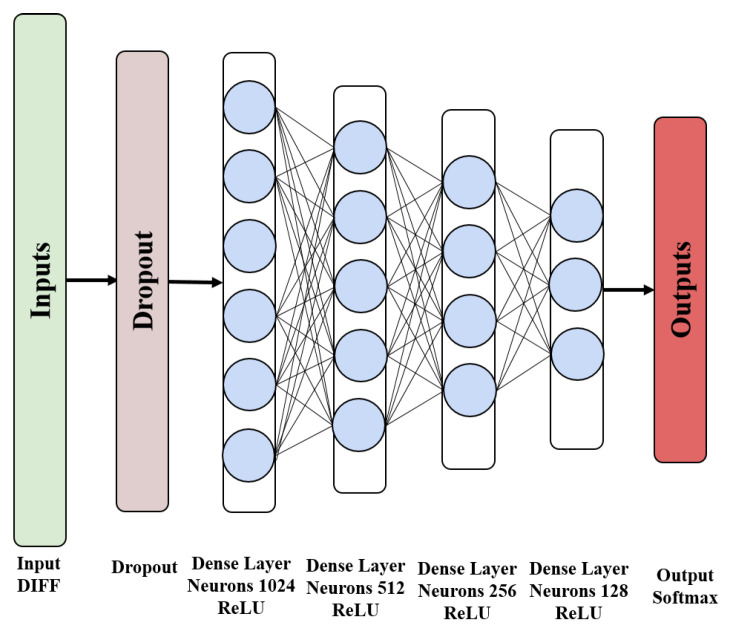
DNN classification model.

In the multi-floor positioning problem, we use an enhanced DNN as the floor positioning model, as shown in Figure 4.

The enhanced DNN model takes DIFF as input data and is trained by the stochastic gradient descent (SGD) algorithm. SGD uses the error back-propagation (BP) algorithm to calculate the loss function between the model prediction and the truth value of the floor. The BP algorithm is composed of two processes, the forward propagation of the signal and the back-propagation of the error. In the forward propagation stage, the DIFF samples enter the DNN from the input layer and are transferred layer by layer to the output layer. If the error between the actual output of the DNN and the expected output is too large, the algorithm turns to the error back-propagation. In the error back-propagation stage, the BP algorithm reversely calculates the error in the DNN, and continuously adjusts the parameters of the neurons θ (weight and bias) in each layer during the back-propagation process to minimize the error. To avoid the disappearance of the gradient, the hidden layer uses the ReLU function as the activation function, and the output layer uses the softmax function defined in (5) to map the input data DIFF to the prediction probability of the real floor to complete the floor classification prediction.

(5) $$\begin{array}{c} \sigma \left(z_{j}\right)=\frac{e^{z_{j}}}{\sum_{k=1}^{K}e^{z_{k}}},j=1,\cdots ,K \end{array}$$

where all the $z_{j}$ values are the elements of the input vector, and any real value can be taken. The term at the bottom of the Formula (5) is the normalization term, which ensures that the sum of all output values of the function is 1, thus constituting a valid probability distribution.

The improved DNN chooses cross-entropy as the loss function. In addition, $L_{2}$ regularization is used to avoid over-fitting. Therefore, the learning of DNN is to minimize the loss function of the parameter set θ in the loss function L(t,y,θ) defined by Formula (6).

(6) $$\begin{array}{c} L\left(t,y,\theta \right)=-\sum_{s=1}^{K}t_{s}logy_{s}+\lambda \sum_{i}\theta_{i}^{2} \end{array}$$

where *t* and *y* represent the target output and the predicted output vectors of the multi-layer perceptron (MLP). The cross-entropy indicates the difference between the amount of information contained in *t* and *y*. If the value of the cross-entropy is smaller, then the predicted output is closer to the target output. The second term of the Formula  (6) is the regular term $\lambda \sum_{i}\theta_{i}^{2}$, where λ is the regularization parameter that needs to be optimized, and the value range is [0, +∞), and $\sum_{i}\theta_{i}^{2}$ represents the sum of squares of the weight parameters.

### 3.4. Sepcific-Location Positioning Model

**Figure 5:**
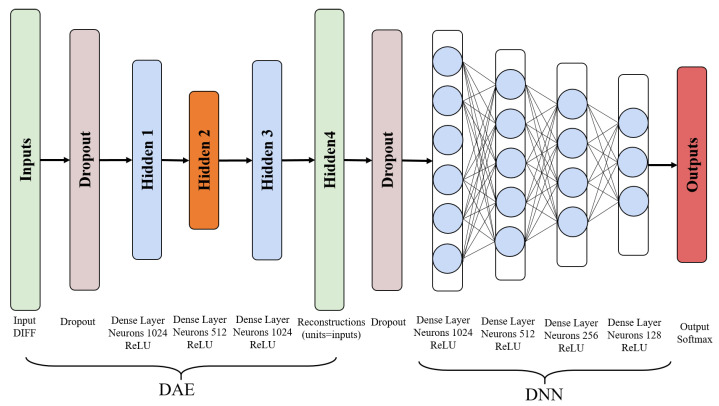
DDAE Location Estimation Model.

After completing the positioning of the building and floor of the mobile user, we need to further locate the specific location of the mobile user. To this end, we design a new DDAE model, as shown in Figure 5. The DDAE model consists of two parts: improved DAE and enhanced DNN. The improved DAE increases the number of hidden layers to 4. At the same time, during the model training process, an improved gradient descent method called adaptive moment estimation (Adam) is used to update the parameter set θ (weights and biases) of the model. Algorithm 1 gives the pseudo-code of DDAE offline training. The Input includes the DIFF value of all training sets, location labels, max_epoch, dropout rate, and learning rate. Firstly, the algorithm initializes all weights and biases randomly (step 1). Then for each epoch, the algorithm randomly selects a mini_batch from the DIFF values of all training sets and feeds these data to the DDAE (step 3). The first and last layers of the DDAE are the input layer and the output layer, respectively. Starting from the second layer, the input data is processed by the dropout layer and the hidden layers (steps 6–17). Step 19 uses the loss function to measure the error between the true position of the training set and the output value of DDAE.
**Algorithm 1** DDAE Weight Training Algorithm**Input:** DIFF value, network architecture, max_epoch, dropout_rate *p*, and learning rate α; **Output:** Trained weights ω and *b*; 1: Randomly initialize ω and *b*; 2: **while** epoch<Max_epoch **do** 3:    Randomly select a mini-batch from inputs; 4:    // Forward propagation; 5:    // *L* is the number of layers of the DDAE; 6:    **for** *l* = 2:L-2 **do** 7:      **if** the current layer is a dropout layer **then** 8:         $r_{j}^{\left(l\right)}∼Bernoulli\left(p\right)$ 9:         ${\tilde{y}}^{\left(l\right)}=r^{\left(l\right)}\cdot y^{\left(l\right)}$ 10:       $z_{i}^{\left(l+1\right)}=\omega_{i}^{\left(l+1\right)}{\tilde{y}}^{\left(l\right)}+b_{i}^{\left(l+1\right)}$ 11:       $y_{i}^{\left(l+1\right)}=f\left(z_{i}^{\left(l+1\right)}\right)$ 12:      **else** 13:         // The current layer is a hidden layer; 14:         $z^{\left(l\right)}=\omega^{\left(l\right)}y^{\left(l\right)}+b^{\left(l\right)}$ 15:         $y^{\left(l\right)}=f_{l}\left(z^{\left(l\right)}\right)$ 16:      **end if** 17:    **end for** 18:    //Loss function; 19:    $Loss=-\sum_{i=1}^{output\_size}y_{i}\cdot log\hat{y_{i}}$ 20: **end while**


## 4. Experimental Setup

This section mainly introduces the UJIIndoorLoc data set used in the experiment and describes in detail the environment in which the data set is collected, as well as the attributes and characteristics contained in this data set. Next, it introduces the core process of data preprocessing and calibration-free processing. Finally, the evaluation indexes used in the experiment are presented.

### 4.1. UJIIndoorLoc Dataset

In the literature related to indoor positioning, UJIIndoorLoc is the comprehensive and accessible data set in the UCI machine learning repository [28]. This data set contains the Wi-Fi RSSI data used in the IPIN 2015 EvAAL competition and is publicly available [41]. The collected data comes from 25 different Android devices with the help of 20 volunteers. Specifically, the data set consists of 21,048 Wi-Fi RSSI data, which are divided into 19,937 training samples and 1,111 verification samples. These samples are collected in the buildings of the University of Jaime I in Spain, covering an area of nearly 108,703 square meters. Each collection in the data set contains 529 attributes. In the data collection area, 520 distinct APs are found, so the first 520 attributes are expressed as the RSSI received from these APs. The signal strength ranges from −104 dBm to 0 dBm. When an AP is unavailable, its signal strength value is 100. The remaining 9 attributes are the latitude and longitude of the measurement point, floor number, building ID, space ID, relative location, volunteer ID, phone ID, and the measurement time stamp.

### 4.2. RSSI Database Optimization

RSSI needs to be optimized before DIFF calibration-free processing to improve positioning accuracy. After analysis, it is found that when the AP is unavailable, its RSSI value is marked as 100, while the available RSSI values range from −104 dBm to 0 dBm. The unavailable APs are identified and deleted. At the same time, the APs are sorted according to the strength of their RSSI, and then the RSSI with stronger signal strength is preferentially selected for positioning.

DIFF brings an explosion of RSSI data dimensions, expanding the dimension size from *m* to $C_{m}^{2}$, which reduces the positioning efficiency. For example, if the 520 optimized RSSI data are directly processed by DIFF, the data dimension will be extended to 134,940. When the positioning algorithm reads the high-dimensional DIFF data, there will be a problem of insufficient memory, resulting in the low running efficiency of the algorithm. In the following experimental section, we analyze this issue in detail. To this end, we design a data hierarchical processing strategy. Specifically, on the basis of the above RSSI optimization process, we first subdivide the DIFF data set into three sub-datasets according to buildings. When positioning, the positioning sequence is from multi-building to multi-floor, and then to the specific location. The experimental results show that this is an effective treatment strategy for high-dimensional input data.

### 4.3. DIFF Calibration-Free Processing

**Figure 6:**
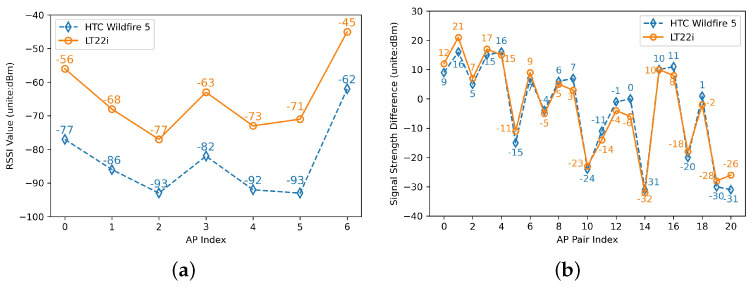
DIFF Calibration-free Processing. (**a**) Signal strength detected by HTC Wildfire 5 and LT22i smartphones at a fixed location. (**b**) Extract signal strength difference from RSSI.

The heterogeneity of wireless receiving devices, co-channel interference, and multi-path effects make the RSSI of Wi-Fi fluctuate greatly. For example, Figure 6a shows the variation curves of RSSI values measured by two different devices at the same location randomly extracted from the RSSI optimization data set.

As shown in Figure 6a, the trends of the two curves are the same, but the values are quite different. According to the logarithmic distance loss model, the value of RSSI is inversely proportional to the distance. Therefore, at the fixed position, the RSSI values of different devices all reflect the distance from the fixed point to the AP [21]. On the other hand, by analyzing the shape of the two curves, it is found that the changing trend of the curves can be represented by the RSSI difference between APs. Therefore, we can extract the RSSI difference of AP pairs to replace the original RSSI value [26].

Assuming that there are *m* APs $R=r_{1},r_{2},\cdots ,r_{m}$, the RSSI value of the APs received at a specific location can be defined as a row vector $s=(s_{1},s_{2},\ldots ,s_{m})$, where $s_{i}$ represents the RSSI value received from AP $r_{i}$. Define the DIFF of an unique AP pair $b_{i}\times b_{j}\in R\times R$, and constrain i<j to be unique. The DIFF *d* can be expressed as:

(7) $$\begin{array}{c} d\left(r_{i},r_{j}\right)=s_{i}-s_{j}\;1\leq i<j\leq m \end{array}$$

Therefore, the obtained DIFF feature vector *D* is expressed as:

(8) $$\begin{array}{c} D=(d\left(r_{1},r_{2}\right),d\left(r_{1},r_{3}\right),\cdots ,d\left(r_{m-1},r_{m}\right)) \end{array}$$

where the length of *D* is *t*, $t=C_{m}^{2}$.

For example, in Figure 6a,

$$S_{HTCWildfire5}=\left(-77,-86,-93,-82,-92,-93,-62\right)\;S_{LTzzi}=\left(-56,-68,-77,-63,-73,-71,-45\right)$$

so,

$$D_{HTCWildfire5}=(9,16,5,15,16,-15,7,-4,6,7,-24,\;-11,-1,0,-31,10,11,-20,1,-30,-31)\;D_{LTzzi}=\left(12,21,7,17,15,-11,9,-5,5,3,-23,-14,-4,-6,-32,10,8,-18,-2,-28,-26\right)$$

As shown in Figure 6b, the difference in features extracted by the two different devices is much more similar than the RSSI value.

### 4.4. Evaluation Index

In multi-building and multi-floor positioning, we use target hit accuracy to evaluate the efficiency of algorithms. In location-specific positioning, we use the average positioning error to evaluate the positioning accuracy of the model. The average positioning error is based on the root mean square error (RMSE), which is defined in the calculation Formula (9) as follows.

(9) $$\begin{array}{c} RMSE=\sqrt{\frac{1}{N}\sum_{n=1}^{N}{\left(x_{n}-\tilde{x_{n}}\right)}^{2}+{\left(y_{n}-\tilde{y_{n}}\right)}^{2}} \end{array}$$

where $\left(x_{n},y_{n}\right)$ represents the real coordinate location of the positioning user, $\left(\tilde{x_{n}},\tilde{y_{n}}\right)$ represents the location coordinates calculated by the positioning algorithm, and *N* represents the number of the calculated positioning coordinates.

## 5. Performance Evaluation

In this section, we evaluate the proposed positioning algorithms by comparing their performance with existing positioning methods. All experiments in this paper are implemented by programming software under the 64-bit Windows 10 operating system. The main computer hardware parameters are 16 GB memory, Intel Core i5-9500 processor with 3.00 GHz, and NVIDIA GeForce GTX 1660 Ti computing card. We use Python-3.6.2 to program software, and train the deep learning model based on Keras-2.3.1 and Tensorflow-GPU-1.14.0.

### 5.1. Model Optimization

We optimize the positioning algorithms through multiple experiments. The final optimized parameters are shown in Table 1. The activation functions in the DAE and DNN algorithms are both corrected linear units (ReLU). The optimizer is Adam with a learning rate of 0.001. The loss function is categorical_crossentropy. The activation function of the output layer is Softmax, and the batch training set is 500.

The choice and setting of the optimizer is a key task in training a DNN. To obtain the optimal network model, for the evaluation indexes in Section 4.4, we evaluate different optimizers and learning rate combinations through experiments. Evaluated optimizers include Adam, Nadam, RMSprop, and AdaMax [42]. The experimental results are shown in Table 2.

The experimental results show that the Adam optimizer has outstanding performance. Furthermore, combined with the optimization of the learning rate, optimal positioning results can be obtained. When the learning rate of the Adam optimizer is 0.005, in the multi-building environment, the building hit rate is 100%. When the learning rate of the Adam optimizer is 0.001, the floor hit rate reaches 96.22%. When the learning rate of the Adam optimizer is 0.001, the positioning error of the positioning estimation is the smallest, which is reduced to 6.01 m.

### 5.2. Performance Analysis of Building and Floor Positioning Models

To evaluate the positioning accuracy of the proposed algorithms in multi-building and multi-floor scenarios, we compare them with traditional machine learning algorithms and current advanced deep learning algorithms, respectively. The experimental results show that our algorithms have obvious advantages in multi-floor and multi-floor positioning scenarios, whether compared with traditional machine learning algorithms or deep learning algorithms. The specific analysis is as follows.

Machine learning algorithms used for comparison include SVM [27], KNN [28], RF [29], DT [30], and GNB [31]. The experimental results are shown in Table 3.

**Figure 7:**
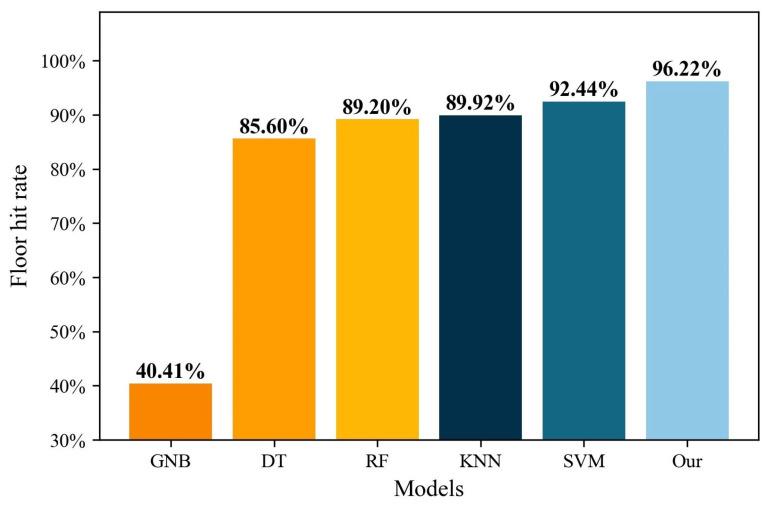
Comparison of our algorithm and machine learning methods for multi-floor positioning.

It can be seen from Table 3 that the hit rate of machine learning methods in building positioning is 97.03% at the lowest (DT) and 99.91% at the highest (RF), while the DAE model proposed in this paper reaches 100%. This indicates that the improved DAE model can effectively handle the multi-building positioning issue. Figure 7 shows the result of multi-floor positioning. As can be seen from Figure 7, in terms of multi-floor positioning, the hit rate of machine learning methods is 40.41% at the lowest (GNB), and 92.44% at the highest (SVM), while the DNN model proposed in this paper reaches 96.22%. The enhanced DNN model greatly improves the multi-floor positioning accuracy.

**Figure 8:**
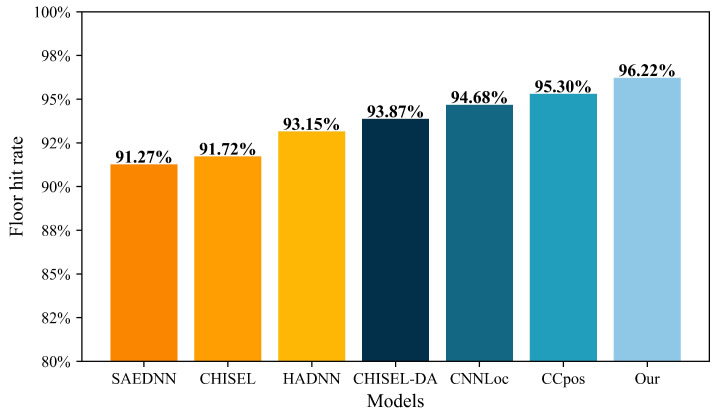
Comparison of our algorithm and state-of-the-art deep learning methods for multi-floor positioning.

To further evaluate the performance of our improved DAE and enhanced DNN algorithms, we compare them with state-of-the-art deep learning indoor positioning algorithms, including SAEDNN [34], CNNLoc [35], CCpos [36], CHISEL [37], CHISEL-DA [37], and HADNN [38]. The results are shown in Table 4 and Figure 8, respectively.

Table 4 shows the results of the above-mentioned indoor positioning technologies in multi-building positioning. It can be seen from Table 4 that the hit rate of deep learning methods in building positioning is 96.03% at the lowest (CNNLoc) and 100% at the highest (HADNN), meanwhile, the DAE model proposed in this paper reaches 100%. This indicates that the improved DAE model can effectively handle the multi-building positioning issue.

Figure 8 shows the results of the above-mentioned deep learning technologies in multi-floor positioning. As can be seen from Figure 8, in terms of multi-floor positioning, the hit rate of deep learning methods is 91.27% at the lowest (SAEDNN), and 95.30% at the highest (CCpos), while the enhanced DNN model proposed in this paper reaches 96.22%. The enhanced DNN model greatly improves the multi-floor positioning accuracy.

### 5.3. Performance Analysis of Specific-Location Positioning Model

In order to evaluate the average positioning error of the proposed DDAE in the specific-location scenario, we compare DDAE with traditional machine learning algorithms and current advanced deep learning algorithms. The experimental results show that DDAE has obvious advantages with the smallest average positioning error in the specific-location positioning scenario, whether compared with traditional machine learning algorithms or deep learning algorithms. The specific analysis is as follows.

Machine learning algorithms used for comparison include SVM [27], KNN [28], RF [29], DT [30], and GNB [31]. Table 5 shows the comparison results of DDAE and the traditional machine learning algorithms.

**Figure 9:**
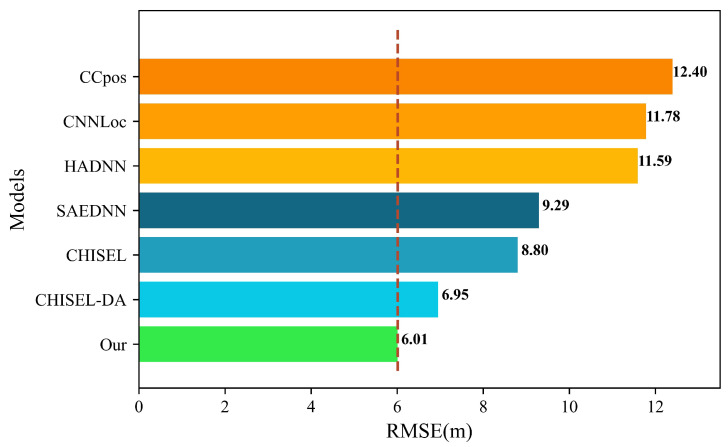
Specific-location positioning error comparison results.

It can be seen from Table 5 that the average positioning error of machine learning methods in specific-location positioning is 6.13 m at the lowest (KNN) and 10.42 m at the highest (DT and GNB), while the DAE model proposed in this paper reduces to 6.01 m. Similarly, the state-of-the-art deep learning algorithms used for comparison include SAEDNN [34], CNNLoc [35], CCpos [36], CHISEL [37], CHISEL-DA [37], and HADNN [38]. Figure 9 shows the comparison results of DDAE and the advanced deep learning algorithms. It can be seen from Figure 9 that the average positioning error of machine learning methods in specific-location positioning is 6.95 m at the lowest (CHISEL-DA) and 12.40 m at the highest (CCpos), while the DAE model proposed in this paper reduces to 6.01 m.

## 6. Conclusions

In this paper, we propose calibration-free indoor positioning algorithms based on DNN and DIFF, which extend the indoor positioning from a single plane to three dimensions, namely, multi-building, multi-floor, and specific location. However, DIFF brings the explosion of data dimensions, which reduces the positioning efficiency. Therefore, we design a data hierarchical processing strategy based on building-floor-specific location, which effectively improves the efficiency of high-dimensional data processing. Aiming at the issue of positioning accuracy, we design an improved DAE and an enhanced DNN to improve the positioning accuracy of multi-building and multi-floor, respectively. In the specific-location positioning scenario, a newly designed positioning algorithm called DDAE is proposed. The DDAE consists of an optimized DAE and an enhanced DNN. By utilizing the optimized DAE to reduce the dimensions of input data DIFF while retaining the key features and optimizing the structure and parameters of enhanced DNN, the DDAE can obtain better positioning accuracy. Our work addresses both positioning accuracy and positioning efficiency issues. Future works may further consider the adaptability of the proposed algorithms. In addition, the construction of a new fingerprint signal data set to verify the performance of algorithms more conveniently and objectively also needs further consideration.

## Figures and Tables

**Table 1:** Model parameter settings.

Parameter	Value
**DAE activation function**	ReLU
**DAE Optimizer**	Adam (lr = 0.001)
**DAE loss**	categorical_crossentropy
**MLP activation function**	ReLU
**MLP Optimizer**	Adam (lr = 0.001)
**MLP loss**	categorical_crossentropy
**Output layer activation function**	Softmax
**Batch_size**	500

**Table 2:** Positioning performance of different learning rates in multi-building, multi-floor, and location estimation.

Optimizer	Learning Rate/%	Building Hit Rate/%	Floor Hit Rate/%	Positioning Error/m
Adam	0.005	**100**	96.13	9.80
	0.001	99.91	**96.22**	**6.01**
	0.0005	99.73	95.95	6.60
	0.0001	99.91	96.13	7.61
Nadam	0.005	99.91	95.05	26.49
	0.001	99.64	96.13	6.47
	0.0005	99.46	95.95	6.31
	0.0001	99.73	95.59	8.63
RMSpro	0.005	98.47	95.05	49.73
	0.001	98.11	95.68	7.37
	0.0005	98.20	95.68	7.06
	0.0001	99.55	95.86	6.60
AdaMax	0.05	99.55	82.63	51.59
	0.01	98.83	95.77	7.47
	0.005	99.37	96.13	7.07
	0.001	99.19	95.50	6.83

**Table 3:** Comparison of our algorithm and machine learning methods for multi-building positioning.

Multi-Building Positioning Model	DT	GNB	KNN	SVM	RF	Our
**positioning accuracy (%)**	97.03	99.19	99.46	99.72	99.91	100

**Table 4:** Comparison of our algorithm and state-of-the-art deep learning methods for multi-building positioning.

Multi-Building Positioning Model	CNNLoc	CCpos	CHISEL	SAEDNN	CHISEL-DA	HADNN	Our
**positioning** **accuracy (%)**	96.03	99.60	99.64	99.82	99.96	100.00	100.00

**Table 5:** Specific-location positioning results.

Specific-Location Positioning Algorithm	DT	GNB	SVM	RF	KNN	Our
**RMSE (m)**	10.42	10.42	8.65	7.14	6.13	6.01

## Data Availability

Not applicable.
